# Identification, Evaluation and Prioritization of Chemicals for National Human Biomonitoring Program: Insights from Latvia

**DOI:** 10.3390/toxics13020096

**Published:** 2025-01-26

**Authors:** Linda Matisāne, Lāsma Akūlova, Žanna Martinsone, Ilona Pavlovska, Laura Komarovska, Kristiāna Venžega, Dace Jakimova, Kristīne Sproģe, Normunds Kadiķis, Inese Mārtiņsone, Madlen David, Marike Kolossa-Gehring, Ivars Vanadziņš

**Affiliations:** 1 Institute of Occupational Safety and Environmental Health, Rīga Stradiņš University, Dzirciema 16, LV-1007 Riga, Latvia; linda.matisane@rsu.lv (L.M.); zanna.martinsone@rsu.lv (Ž.M.); dace.jakimova@rsu.lv (D.J.); normunds.kadikis@vi.gov.lv (N.K.); ivars.vanadzins@rsu.lv (I.V.); 2 Laboratory of Hygiene and Occupational Diseases, Institute of Occupational Safety and Environmental Health, Rīga Stradiņš University, Dzirciema 16, LV-1007 Riga, Latvia; ilona.pavlovska@rsu.lv (I.P.); laura.komarovska@rsu.lv (L.K.); kristiana.venzega@rsu.lv (K.V.); kristine.sproge@rsu.lv (K.S.); inese.martinsone@rsu.lv (I.M.); 3 Health Inspectorate of the Republic of Latvia, Klijanu Street 7, LV-1012 Riga, Latvia; 4 German Environment Agency, Corrensplatz 1, P.O. Box 33 00 22, 14195 Berlin, Germany; madlen.david@uba.de (M.D.); marike.kolossa@uba.de (M.K.-G.)

**Keywords:** human biomonitoring, national program, chemical prioritization, adaptation of Hanlon methodology

## Abstract

Human biomonitoring (HBM) is a critical tool for assessing chemical exposure in populations and informing public health policies. This study aimed to prioritize chemical substances for the development of a national HBM program in Latvia, addressing the need for systematic evaluation of chemicals in the local context. Initially, 318 chemical substances were reviewed, of which 130 were shortlisted and assessed using an adapted Hanlon methodology. Substances were assessed based on their health significance, hazardous properties, exposure characteristics, national relevance, and public interest. The results identified 30 high-priority substances across various categories, providing a foundation for the HBM4LV program. This prioritization process highlighted the challenges of data gaps, resource limitations, and the need to balance national priorities with alignment to European frameworks. Despite addressing key methodological challenges, the study highlights the importance for ongoing refinement, robust data collection, and strengthened international collaboration to enhance the program’s scope and long-term sustainability. While the methodology addressed key challenges, further refinement and international collaboration are essential to enhance the program’s scope and sustainability.

## 1. Introduction

Human exposure to chemicals is a global health concern because everybody is exposed to chemicals in their daily lives, as different substances are present in the air, water, soil, consumer products, and workplaces. According to the World Health Organization, human biomonitoring (HBM) is a method which is used to assess human exposure to chemicals by measuring their concentration in human body fluids or tissues, such as blood or urine [1]. HBM programs allow evaluation of whether chemical detection rates or concentration values in a group or population are harmful and whether action is needed to reduce the risks from exposure [1].

Planning and implementing national HBM programs require broad expertise alongside substantial financial and personnel capacities. HBM as a systematic approach for management of chemical substances at workplaces was initiated at the beginning of the 20th century, but the concept of HBM started in the 1970s and 1980s in the USA and Germany, respectively [2]. In the 19th century, industrialization increased chemical exposure in workplaces, and early monitoring focused on identifying poisoning symptoms rather than measuring chemicals in biological samples [3]. Existing national HBM programs provide governments, experts (health experts, experts on the environment, food safety experts, manufacturers, producers in agriculture, scientists, etc.), and citizens with evidence-based and time-trend data on chemical exposures and are a reliable source of information for awareness-raising activities and policy changes. Outcomes of existing HBM programs confirm the usefulness of the national- and global-level data on the presence of chemical compounds in the environment and the human body, the time-trend of chemicals based on their use in production, restrictions, etc., as well as health outcomes after identified exposure levels. Globally, there are many countries with well-established HBM systems, e.g., the USA (National Health and Nutrition Examination Survey) [4], Canada (Canadian Health Measures Survey) [5,6], and South Korea (the Korean National Environmental Health Survey (KoNEHS) [7]. Several EU countries (e.g., Germany, Belgium, France) also run their own national HBM programs, and the obtained data are used for setting and evaluating control measures, monitoring trends of exposure and new/emerging exposures, and informing the population on chemical risks to human and environmental health [8].

So far, only a few sporadic HBM studies have been conducted in Latvia. The available data from these studies are not comprehensive enough to provide a detailed evaluation of chemical exposure across the population, as the previous attempts to carry out HBM studies were primarily project-based and, therefore, focused on a narrow area, for example, the exposure to persistent organic pollutants in breast milk [9]. Some non-governmental organizations have also carried out research on exposure to plastics [10] and house dust samples, unfortunately lacking a properly elaborated methodology [11]. To the best of our knowledge, there is only one well-organized, scientific HBM study (but with limited scope and resources) that was carried out under the project “Human Biomonitoring for Europe” (HBM4EU) [12,13]. Co-funded by HORIZON2020, HBM4EU was a joint effort of 30 countries, the European Environment Agency, the European Commission, and national authorities, aimed at the evaluation of actual exposure of citizens to support policy making and create new evidence.

At the end of 2023, the Ministry of Health initiated a state-financed national research programs project, “Development of Human Biomonitoring program for Latvia (HBM4LV)”, which aims to create an evidence-based background for the Latvian Human Biomonitoring program and to describe the first steps to implement the national HBM program related to pesticides, heavy metals, and certain organic pollutants in practice. A running HBM program would improve the prevention of many diseases and health conditions resulting from environmental, food, or household chemical exposure, ensure a sentinel system for new and emerging risks and potential health problems, and allow monitoring of several exposure-linked public health indicators [14].

The national HBM program requires a vast amount of resources, therefore, the final decision on chemical compounds that are monitored depends not only on chemical substances/chemical substance groups, their metabolites, or other markers but also on the recruiting and sampling strategies as well as data linkage with other health studies. It also highly depends on the availability of funds [15]. Therefore, a prioritization strategy has to be used. Several available methods for priority setting are used for public health aspects and HBM. For example, different prioritization criteria for national HBM programs are used in the United States National Health and Nutrition Examination Survey, the Canadian Health Measures Survey, the German Environmental Survey, the French Longitudinal Study of Children, the French cross-sectional health survey, and the Flemish Environment and Health Study [16].

At the European Union (EU) level, a prioritization of chemicals for HBM has been recently performed by the HBM4EU project [16], consisting of several steps. The first step was to map knowledge needs and initiate the prioritization; the second step was to rank nominated substances/substance groups from the shortlist; the third step consisted of consulting with the EU Policy Board and the HBM4EU Management Board to agree on a list of proposed priority substances [16]. However, the experts’ task in Latvia was more complicated than reusing the list prepared by the HBM4EU project and aligning it with national needs. This was because the use of the well-known Hanlon method was predefined by the national authorities (the Ministry of Health).

The Hanlon method is one of the most commonly used systems for prioritizing public health problems [17]. It was first described in 1954 and subsequently improved by adding an equation that calculates a basic priority rating score for ranking health problems [18,19]. The Hanlon method or the basic priority rating system prioritizes issues by assigning a score (from 1 to 10) in each of the following categories: magnitude (size), importance (urgency, severity, consequence), and potential intervention success [17]. However, this method does not always satisfy all of the needs (e.g., regional differences related to the prevalence of different diseases) resulting in amendments made by different organizations to tailor the approach to their specific requirements [20]. For example, the Pan American Health Organization refined the original approach by making it suitable for prioritizing public health programs that include disease and non-disease control areas. It used the following components: (1) component A (size of the problem); (2) component B (seriousness); (3) component C (effectiveness); (4) component D (PEARL); and (5) component E (inequity) [20].

Since one of the initial tasks of the HMB4LV project was to provide evidence for decision making on the prioritization of chemicals, an approach was designed using an adaptation of the Hanlon method. This article thoroughly describes the approach used in Latvia—covering the adaptation of the Hanlon method to the national context, the entire prioritization process, and the resulting list of assessed chemical substances and chemical substance groups, as well as the final result, the list of prioritized substances and their groups. We believe that this approach suits the needs of a country yet to run a national HBM program.

## 2. Materials and Methods

**Figure 1:**
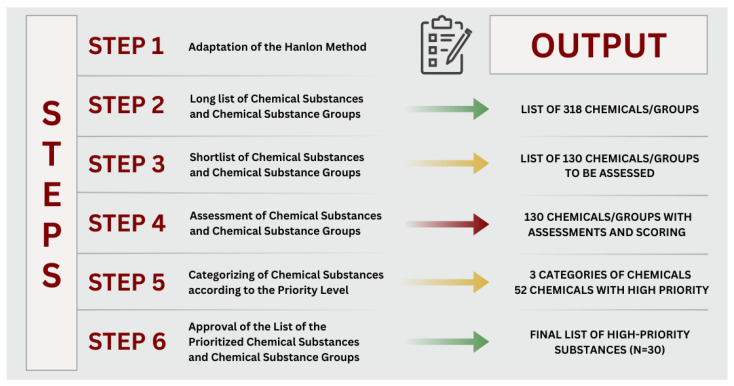
Six-step approach for prioritization of chemicals/groups of chemicals.

In general, we used a similar approach to other groups of researchers involved in the prioritization of chemicals for HBM purposes [16] in the past. A structured six-step approach was used (for details, see Figure 1).

First (step 1), a literature review on the prioritization strategies and methods was carried out to collect methods and criteria used for chemical prioritization in HBM programs worldwide. Based on the literature search and the Hanlon method which was required by the national authorities, we created and validated a tailored tool to match the needs for the national HBM program in Latvia. Step 2 was devoted to preparing the long list of chemicals and their metabolites to be ranked. For this purpose, another thorough literature search was conducted, and additional information was gathered. Furthermore, we invited ten state authorities working in health or environmental fields to nominate substances/substance groups that should be prioritized based on their expertise. Then, in step 3, a shortlist of chemicals was made based on suggestions from the authorities and a literature review. Step 4 was the most demanding and time-consuming. The assessment according to the adapted Hanlon method was carried out for each shortlisted chemical substance and group of substances. After that, all chemical substances were ranked into four independent groups (three groups were defined by the Ministry of Health—pesticides, heavy metals, and persistent organic pollutants [14]—the fourth was added by researchers based on the literature review and analysis of other national HBM programs and covered all other chemicals not included in the above-mentioned three groups). During step 5, all of the chemicals were prioritized into four groups according to the calculated points (high priority, average priority, low priority, no priority), and this information was included in the lists prepared earlier. These lists, together with the supporting documents, were submitted to the Human Biomonitoring Council of Latvia for review for agreement on the final list of proposed priority substances and substance groups and the approval of the list. This was step 6. Minor document changes were made after receiving comments from the Human Biomonitoring Council of Latvia. The entire process took place between December 2023 and November 2024 when the Human Biomonitoring Council approved the list of the prioritized chemical substances and chemical substance groups.

### 2.1. Step 1—Adaptation of the Hanlon Method

The adaptation of the Hanlon method was performed by an expert panel with a background in chemistry, public health, medicine, and the environment. The adapted Hanlon equation was used for the prioritization of the chemical substances/chemical substance groups to be included in the HBM4LV program. From the original Hanlon equation [17], our adapted method retained component A (problem size), but component B (severity of the problem) is expressed with two components in the adapted version: component B (hazardous properties) and component C (exposure characteristics). For further adaptation to the national context, component D (national significance) and component E (public interest) were added to the equation (Table 1).

To define the scale of the problem (component A), the original Hanlon method used scores between 1 and 10, where 1 point is exposure of <0.01% of the population, 2 points—from 0.01% to 0.09%, etc.). After initial tries to use the original Hanlon equation, we found it challenging to use the 10-point scale because of the type of data evaluated. The original 10-point evaluation system in the Hanlon method seemed less practical and overly complex due to its reliance on highly granular population exposure data, which are often unavailable with such precision in the case of HBM. To address this, the authors adopted a simplified 6-point system, which is more practical, easier to use, and better suited to the scale of available data while maintaining proportionality and effectively prioritizing chemicals (detailed information is provided in Table 1).

Components B and C were evaluated using the GreenScreen^®^ for Safer Chemicals method that has been developed to identify chemicals of high public concern and safer alternatives. It classifies the level of danger of the chemical to human health and the environment through categories [21]. Each component consists of several subcomponents that are evaluated with 1, 3, or 6 points. These points correspond to a high, medium, or low grade (for hazards—component B) or level (for exposure characteristics—component C). In addition, substances with a lack of data were assigned 2 points to account for data gaps, ensuring that substances with insufficient information could still be included in the prioritization process. This adjustable score allowed for a more balanced assessment, acknowledging the uncertainty while maintaining the substance’s potential relevance in the national context.

Component D represents national significance and evaluates whether the substance is monitored within other national programs, such as environmental monitoring or assessing pesticide residues in food. Component D was scored with 0 or 1 point, depending on whether the substance is included in these monitoring programs (No = 0; Yes = 1). This approach ensures that substances already monitored in other contexts are appropriately recognized for their broader significance. A similar principle was used for component E to assess whether or not recent activities of non-governmental organizations were carried out to raise awareness in society of the substance/group of substances, indicating the public’s interest (No activity = 0; There is an activity = 1).

Since components B and C consisted of subcomponents that were evaluated using the approach of the HBM4EU project, a maximum score was calculated for each component. Component B had a maximum score of 60 (10 outcomes, a maximum of 6 points per outcome), while component C had a maximum score of 36 (6 parameters, a maximum of 6 points per parameter). For component B, the following ten outcomes were assessed: (1) carcinogenicity; (2) mutagenicity; (3) reproductive toxicity; (4) developmental toxicity; (5) endocrine activity; (6) systemic toxicity after repeated exposure (STOT RE); (7) neurotoxicity; (8) immunotoxicity; (9) respiratory sensitization; and (10) skin sensitization. For component C, the following parameters were assessed: (1) persistency and/or bioaccumulation potential; (2) sales in the EU or, where possible, sales in Latvia (tonnes per year); (3) exposure routes; (4) passage of placental barrier; (5) exposed population; and (6) level of concern of the exposure. Each outcome/parameter was evaluated according to the “worst-case scenario”, meaning that if at least one of the assessment sections scored 6 points, the overall score was also 6. The worst-case scenario principle was also used for the assessment of chemical substance groups: if the outcome/parameter of an individual substance included in the group was assessed as having 6 points, then the overall result was also 6 points, even if for other substances in the group the outcome/parameter was only 3 or fewer points. The sum of the points was divided by the maximum possible number of points, obtaining a factor. Further, the results obtained for each component were multiplied by the so-called significance weights. The weights were predefined by the researchers and approved by the Human Biomonitoring Council (the membership of the Council is described below). The following weights were used to evaluate substances:

- Hanlon component A—10%;
- Hanlon component B—30%;
- Hanlon component C—30%;
- Hanlon component D—15%;
- Hanlon component E—15%.

Appendix A, Table A1 (acetamiprid, CAS number 135410-20-7) provides an example of using the adapted Hanlon method.

### 2.2. Step 2—Long List of Chemical Substances and Chemical Substance Groups

Identifying prioritized chemical substances/chemical substance groups was carried out in several substeps. First, three priority groups of substances were predetermined in the national research program by the Ministry of Health: pesticides, heavy metals, and persistent organic pollutants [14]. This reflected the research needs that the state has prioritized. Secondly, the identification of specific substances/substance groups within these groups was carried out by the researchers, taking into account the following aspects:

- Results of previously conducted research in the field of HBM worldwide;
- List of priority substances under the HBM4EU project [16];
- Data on hazardous properties of chemical substances/chemical substance groups, exposure characteristics (in particular, potential exposure of the general public);
- Data from the European Food Safety Authority (EFSA) reports, substance infocards, and publications [22];
- Registration dossiers from the European Chemicals Agency [23];
- Reports on the national monitoring programs (e.g., drinking water, food safety, wastewater monitoring, etc.);
- Meeting minutes of the Human Biomonitoring Council of Latvia over the last three years;
- Public opinion/concerns identified through a review of articles on internet news portals and social media over the past five years;
- Searching of the scientific literature (e.g., PubMed);
- Additional criteria specific to the predefined chemical substances/chemical substance groups.

Among the examples of additional criteria used in the case of pesticides, the national data from the previously conducted studies (like the SPECIMEN study) [24] were used. Initially, the researchers recommended including substances (pesticides) detected in the urine samples of at least 15% of the study participants. The Human Biomonitoring Council of Latvia later approved this approach. For pesticides, data on the sales volume of plant protection products distributed in Latvia were used: the assessment was carried out for the substances that were sold in the largest volumes [25].

### 2.3. Step 3—Shortlist of Chemical Substances and Chemical Substance Groups

The shortlisting of chemical substances/chemical substance groups to be prioritized was also carried out in several substeps. Initially, the research team sent letters to the state authorities and other relevant organizations asking them to recommend chemical substances/chemical substance groups or their metabolites that, from the perspective of that particular institution, should be included in the HBM program in Latvia. In addition, these institutions were asked to provide a brief justification for including each substance/substance group. In total, ten answers from the following institutions providing information on chemical substances to be assessed for inclusion in the HBM program of Latvia were received, representing the opinions of eleven organizations:

- the Ministry of Health;
- the Ministry of Agriculture;
- the Ministry of Welfare;
- the Ministry of Environment and Regional Development;
- the Health Inspectorate;
- the State Plant Protection Service;
- the Institute of Food Safety, Animal Health and Environment “BIOR”;
- the Latvian Environment, Geology and Meteorology Centre “SLLC”;
- the Consumer Rights Protection Centre;
- the Food and Veterinary Service;
- the Latvian Medical Association (the opinion was included in the letter from the Ministry of Health).

Then the long list of chemicals was arranged to evaluate which chemical substances/chemical substance groups have been mentioned in the letters of authorities. The list was amended with the missing chemicals. Then the list was shortened (from 318 reviewed chemicals to 130 shortlisted chemicals) by excluding chemical substances/chemical substance groups based on the following criteria:

- the chemical substance/chemical substance group is only mentioned because of the work environment and the legal requirements that require an employer to perform biological monitoring at the workplace level and the particular substance/group is not typically included in national HBM programs (e.g., benzene, acetone, etc.);
- other substances out of the scope of the national research request (e.g., microplastics);
- substances lacking information on hazardous properties regarding the environment and human health provided by ECHA, which made it impossible to perform the assessment based on the Hanlon methodology (e.g., many phthalates).

### 2.4. Step 4—Assessment of Chemical Substances and Chemical Substance Groups

Three different teams of researchers were established to assess chemical substances/chemical substance groups. Each group consisted of at least two researchers with different backgrounds—one of them had a background in chemistry, another one in public health or medicine. All of the researchers had experience in working with the substance group they needed to assess. To reach consistency in assessment, written guidelines on the work process were prepared (in particular, covering the main sources of information and calculation principles). In addition, supporting files were prepared to facilitate the calculation process, and a meeting was organized where each group of researchers participated in a discussion and received a detailed explanation of the adapted Hanlon method.

Following this session, each group independently conducted an evaluation of one specific chemical substance or substance group. Then, all of the researchers met together to discuss the results, amend the possible outputs, and then started to work on all other assessments individually. Reviews and amendments were performed in several stages for each document. The initial draft of each chemical substance/chemical substance group and the assessment documents were reviewed and commented on by a senior researcher and amended according to the received comments. After that, another internal review by a researcher experienced in working with chemical assessments was carried out, and once again, the assessments were amended. After that, the files were proofread, technically edited, and prepared for submission to the Human Biomonitoring Council of Latvia.

### 2.5. Step 5—Categorizing of Chemical Substances According to the Priority Level

For each chemical substance, the total number of points was calculated. In theory, the maximum possible number of points was 186. The classification of substances into priority levels for inclusion in the HBM program in Latvia was guided by predefined cut-offs established through the opinion consensus of the involved researchers, experts, and the members of the Human Biomonitoring Council of Latvia as well as practical considerations like availability of the resources for future testing of samples. These cut-offs were calculated using proportional intervals based on the maximum score, ensuring a consistent and transparent methodology. Depending on the total number of scored points, the substances were classified into the following priority levels for inclusion in the HBM program in Latvia:

- If the number of points was 50% or more of the maximum possible number of points (93 points), the substance was classified as having a high priority for inclusion in the HBM program in Latvia;
- If the number of points ranged from 40 to 49.99% of the maximum possible number of points (74.4 to 92.9 points), the substance was classified as having an average priority for inclusion in the HBM program in Latvia;
- If the number of points was in the range from 30 to 39.99% of the maximum possible number of points (54.9 to 74.39 points), then the substance was classified as having a low priority for inclusion in the HBM program in Latvia;
- If the number of points was up to 29.99% of the maximum possible number of points (or 54.89 points), it was concluded that it is possible not to include the substance in the HBM program in Latvia.

Based on the assessment, chemical substances/chemical substance groups were arranged in a list, starting with the highest total score calculated according to the adapted Hanlon method. The suggested priority level was added to the list. Such ranked lists were made for all four groups separately (pesticides, heavy metals, persistent organic pollutants, and others) (for details, see Appendix A, Table A2). Later, a combined list was created to identify the high-priority substances to be included in the HBM4LV program.

### 2.6. Step 6—Approval of the List of the Prioritized Chemical Substances and Chemical Substance Groups

The lists of ranked chemical substances/chemical substance groups and documents providing gathered information and calculations were submitted to the Human Biomonitoring Council of Latvia which has ten members from ten different organizations (head of the Human Biomonitoring Council is the representative from the Centers for Disease Prevention and Control, other members represent the following organizations: the Ministry of Environmental Protection and Regional Development, the Ministry of Welfare, the Ministry of Education and Science, the Health Inspectorate, Rīga Stradiņš University, Working Groups on Chemicals and Pesticides of the Environmental Advisory Council, the Ministry of Health, the Ministry of Agriculture, Public Health Association of Latvia). These experts had the opportunity to review and suggest amendments to the documents. The Human Biomonitoring Council of Latvia operates using the principle of reaching unanimity. Therefore, discussions were held among the members. The Council suggested several changes, specifically addressing:

1. changing the evaluation scale for sales of pesticides (see below in the next paragraph);
2. using only approved classification, labeling, and packaging (CLP) of chemicals instead of the worst-case scenario and classifications by manufacturers;
3. excluding chemicals with only acute effects and effects after direct contact with skin from the priority list for inclusion in the HBM4LV program.

The evaluation methodology was slightly modified following discussions with the Human Biomonitoring Council of Latvia. Given the wide range in pesticide sales volumes in Latvia (from 0.005 to 504.452 tonnes) [25], it was recommended to adjust the initial scaling system. The original scale was as follows: (1) <10 tonnes scored 1 point; (2) 10–1000 tonnes—3 points; and (3) >1000 tonnes—6 points. The scale was revised to (1) <10 tonnes—1 point; (2) 10–50 tonnes—3 points; and (3) >50 tonnes—6 points. These adjustments influenced the score of Hanlon component C (hazardous properties) and, thus, also the total score for four pesticides: glyphosate, MCPA, metazachlor, and chlormequat chloride.

The assessment documents reflected the final amendments. After that, the agreement on the final list of proposed priority substances among the members of the Human Biomonitoring Council of Latvia was reached.

### 2.7. Use of AI-Assisted Tools in Manuscript Preparation

Portions of this manuscript, including parts of the Discussion, Conclusions, and Abstract, were drafted with the assistance of ChatGPT-4 (OpenAI, San Francisco, CA, USA). While the tool provided support in drafting, its contributions do not meet the criteria for authorship. All content generated with ChatGPT-4 was thoroughly reviewed and edited by the authors to ensure accuracy, alignment with academic standards, and scientific integrity. The authors take full responsibility for the final content of the manuscript.

## 3. Results

### 3.1. General Analysis

Initially, 318 chemical substances were reviewed, from which 130 were shortlisted as described in step 3 of Section 2 and evaluated using the adapted Hanlon methodology:

1. 16 pesticides reviewed and evaluated;
2. 235 persistent organic pollutants reviewed and 55 evaluated;
3. 12 metals reviewed and 9 evaluated; and
4. 55 substances categorized as “other” reviewed and 50 evaluated.

The complete assessment results, including the name of each chemical substance, CAS number, scores (by adapted Hanlon subcategories and total), and priority group, are presented in Appendix A, Table A2. Of these, 52 chemical substances were classified by experts as high-priority candidates for inclusion in the HBM4LV program (Table 2). However, after excluding substances (1) assessed solely based on manufacturer classifications and (2) those with only acute effects or effects limited to direct skin contact, the Human Biomonitoring Council of Latvia approved 30 chemical substances as having high priority for inclusion in the HBM4LV program (Table 3).

The highest total score according to the adapted Hanlon methodology was calculated for one of the pesticides—glyphosate (CAS number 1071-83-6; the total score—145.5)—the second highest for one of the persistent organic pollutants—perfluorooctane sulfonate (CAS number 1763-23-1; the total score—133.8)—and the third highest score for one of the phthalates—bis(2-ethylhexyl) phthalate/Di(2-ethylhexyl) phthalate (DEHP) (CAS number 117-81-7; total score—129.9). The experts suggested including polychlorinated biphenyls (PCB, CAS number 1336-36-3), scoring the fourth highest result (128.4), but the Human Biomonitoring Council of Latvia decided on the exclusion of this group due to lack of CLP classification and suggested further research on this aspect through research projects rather than the national human biomonitoring project. The fifth highest score was calculated for another persistent organic pollutant—benzo(a)pyrene (CAS number 50-32-8; total score—127.8)—which belongs to the polycyclic aromatic hydrocarbons.

### 3.2. Pesticides

In addition to glyphosate (CAS number 1071-83-6), acetamiprid (CAS number 135410-20-7) was also identified as a high-priority substance for inclusion in the HBM program based on its total score of 114.6 points. Several other pesticides received scores ranging from 75.1 to 90.9 points, indicating their classification as average-priority substances for inclusion in the program. These include bentazone, prosulfocarb, MCPA, chlorpropham, and organophosphate pesticides such as dimethoate and pirimiphos-methyl. Most of the evaluated pesticides scored below 70.8 points and were therefore classified as low-priority substances for inclusion in the program.

As described above, we had to adjust the initial scaling system. However, all recalculated scores resulted in the same changes, shifting from 3 points (average level) to 6 points (high level). These revisions did not alter the priority group classification of the pesticides but led to slight increases in total scores: glyphosate increased by 4.8 points (from 140.7 to 145.5), MCPA by 2.4 points (from 80.7 to 83.1), metazachlor by 2.4 points (from 70.8 to 73.2), and chlormequat chloride by 2.4 points (from 69.6 to 72.0).

### 3.3. Persistent Organic Pollutants

In total, 55 chemical substances were assessed using the adapted Hanlon methodology; 17 were categorized as high priority and 5 as average priority for inclusion in the national HBM program (for details, see Appendix A, Table A2). This group is not a unified one and includes eleven flame retardants, seventeen polycyclic aromatic hydrocarbons, five per- and polyfluoroalkyl substances, five polychlorinated biphenyls (PCBs), dioxin-like biphenyls, and biphenyls, seven dioxins, and nine furans. In addition, several chemical substances from PCBs, dioxin-like biphenyls, and biphenyls, which were initially included in the list of chemical substances to be classified, were not included in the list as they scored below 33.5 total points.

The total scores of individual substances included in this group were as diverse as the group itself. The total scores ranged from 133.8 (perfluorooctane sulfonate; CAS number 1763-23-1) to 33.5, the lowest score for persistent organic pollutants included in the total assessment. Despite having high scores, after the decision of the Human Biomonitoring Council of Latvia on the exclusion of chemical substances due to the lack of CLP classification, the following substances were withdrawn from the list: (1) polychlorinated biphenyls (PCBs); (2) indeno(1,2,3-cd)pyrene; (3) benzo(ghi)perylene; (4) anthracene; and (5) pyrene.

### 3.4. Metals

When looking at the total scores of nine assessed metals, only two of them were classified as having a high priority: (1) mercury and (2) lead. Cadmium was classified into the average-priority group, but arsenic and nickel as well as chromium were classified in the low-priority group. Zinc, vanadium, and cobalt scored below the lowest level of inclusion in the lowest-priority group.

### 3.5. Others

When certain substances identified for inclusion in the prioritization list did not fall into any of the previously mentioned categories (pesticides, persistent organic pollutants, and metals), they were classified under the group “others”. The largest subgroups within this category were phthalates, comprising 25 individual substances, and parabens, with 14 individual substances. Acrylamide (CAS number 79-06-1) and triclosan (CAS number 3380-34-5) were assessed as standalone substances. Additionally, the “others” group included bisphenols, encompassing nine distinct bisphenol compounds.

In total, 30 substances were classified as high priority, which is 57.7% out of all high-priority substances. Although all of the assessed phthalates were classified by experts as high-priority substances, 13 substances from this group were excluded from the experts’ suggested list of high-priority substances due to the missing CLP classification. The highest total scores were observed for bis(2-ethylhexyl) phthalate/Di(2-ethylhexyl) phthalate (DEHP), diisobutyl phthalate (DiBP), di-n-butyl phthalate (DnBP), diisohexyl phthalate (DiHP), and dicyclohexyl phthalate (DCHP).

Acrylamide scored 105.7 points and was classified as a high-priority substance, but triclosan had 79.5 points and average priority. Bisphenol A was classified into the group of high-priority substances, but bisphenol S and bisphenol F into the average-priority group. All other bisphenols scored around 40 points or fewer. Therefore, they were not suggested for inclusion in the national HBM program. All parabens were excluded from the list of substances with high priority due to the missing CLP classification.

## 4. Discussion

The prioritization of chemical substances for the HBM4LV program represents a significant achievement in establishing a national framework for HBM in Latvia. By adapting the Hanlon methodology, this study successfully incorporated local priorities, resource constraints, and public health considerations into a comprehensive assessment process [17]. The results highlighted 30 high-priority substances, addressing the dual objectives of reflecting Latvia’s specific needs and aligning them with European and global initiatives such as the HBM4EU and the European Partnership for the Assessment of Risks from Chemicals (PARC) frameworks [16]. This alignment enhances Latvia’s capacity to contribute to collective efforts in monitoring chemical exposures and protecting public health while maintaining regional relevance. For example, the United States’ National Health and Nutrition Examination Survey [4] focuses on a wide range of chemicals, including heavy metals, pesticides, and persistent organic pollutants, many of which overlap with Latvia’s high-priority list. Similarly, Canada’s Canadian Health Measures Survey [5,6] emphasizes chemicals with significant health impacts, including phthalates and parabens, which are also prominent in the Latvian prioritization. European countries such as Germany, Belgium, and France have well-established HBM programs that monitor similar chemical groups, particularly persistent organic pollutants and heavy metals, reflecting shared concerns across EU member states [8]. In South Korea, the Korean National Environmental Health Survey [7] targets regionally relevant chemicals, including pesticides and industrial pollutants, mirroring the inclusion of pesticides and persistent organic pollutants in Latvia’s list. While Latvia’s prioritization methodology incorporates unique national and regional exposure patterns, the resulting list demonstrates significant alignment with international programs, facilitating potential collaboration and data comparability across borders.

The integration of public interest and national significance as key components of the adapted Hanlon methodology ensured that societal concerns were adequately reflected in the rankings [26]. These factors added depth to the prioritization process, capturing not only the scientific and regulatory aspects but also the broader implications for public awareness and policy making. Furthermore, this approach demonstrated the adaptability of the Hanlon method to contexts beyond its original public health applications, offering a model for other nations embarking on similar initiatives [20].

However, achieving consensus among experts posed notable challenges. Diverging perspectives on evaluation criteria—particularly regarding pesticide usage—complicated the process. Some experts advocated for metrics based on total sales volumes within Latvia or Europe, while others emphasized usage per hectare of specific crops [25]. These differences necessitated iterative adjustments to scoring thresholds, including the revision of tonnage categories to better reflect Latvia’s agricultural practices. Similarly, the inclusion of substances with limited data, such as those lacking CLP classifications, required reliance on self-reported manufacturer data or findings from scientific literature [23]. These supplementary sources, while valuable, introduced variability in the assessment process and highlighted the need for more robust regulatory frameworks to ensure consistent data availability.

The classification process also underscored the importance of harmonizing national and international priorities [27]. While the majority of identified substances align with European efforts, some regional differences in exposure scenarios required tailored adjustments. For example, locally observed patterns of pesticide application and pollutant distribution influenced the rankings, emphasizing the importance of flexibility in adopting global frameworks. The robust discussion among experts ultimately led to a methodology that balances scientific rigor with practical applicability, ensuring that the HBM program serves as both a public health tool and a model for collaborative decision making.

The successful prioritization of chemical substances provides a solid foundation for implementing the HBM4LV program, but further efforts are needed to achieve its full potential [16]. First, the development of detailed protocols for sampling, data collection, and analysis is essential to ensure consistent and reliable monitoring. These protocols must be tailored to the specific properties of the high-priority substances identified in this study. Collaboration with international initiatives, such as PARC and HBM4EU, will play a critical role in refining methodologies, sharing expertise, and enhancing data comparability. Addressing data gaps, particularly for substances with limited exposure or toxicity information, will require targeted research and better integration of global databases.

To ensure the feasibility and effectiveness of the HBM program, a pilot study is planned involving 30 participants. This pilot will focus on testing of all high-priority chemicals or their metabolites to assess the practical aspects of sampling, detection, and analysis. The results of this pilot study will provide critical insights into the monitoring process and may lead to amendments to the current list of prioritized substances, ensuring that it remains relevant and evidence-based. This phased approach allows for the refinement of the monitoring framework before broader implementation, ensuring that resources are utilized efficiently while maintaining alignment with national and European priorities.

This study faced several limitations that underscore the need for ongoing refinement of the HBM4LV program. One of the primary challenges was the availability of comprehensive and high-quality data, particularly for emerging substances and those without established CLP classifications. This led to reliance on alternative sources such as self-reported classifications, expert judgment, and findings from scientific literature, which, although useful, introduced variability in the scoring process. The variability in data availability across chemical categories also posed difficulties in ensuring a consistent evaluation process, particularly for persistent organic pollutants, like phthalates, and parabens.

Resource constraints further limited the scope of this analysis, restricting the inclusion of additional substances or the application of more extensive validation steps. The adapted Hanlon methodology, while effective in addressing national priorities, may not fully align with international needs. This misalignment could present challenges for cross-country comparability, limiting Latvia’s ability to benchmark its progress against other nations with established HBM programs. Another key limitation was the lack of a clearly defined and standardized strategy for national prioritization in other countries. While frameworks such as HBM4EU and PARC provided general guidance, these were primarily focused on broader European priorities rather than the specific needs of smaller nations like Latvia. This gap required the Latvian team to develop and adapt its methodology independently, which, while successful, involved significant reliance on expert judgment and iterative adjustments.

As adapted for this study, the Hanlon methodology provides a structured and transparent framework for prioritizing chemical substances; however, it is not without limitations. One key challenge lies in its reliance on available data to inform scoring, which may lead to the exclusion of emerging chemicals with limited toxicological or exposure information. Despite efforts to incorporate a wide range of substances through literature reviews and consultations, the evolving nature of chemical production and use introduces a risk of overlooking newly identified substances. Another limitation pertains to the ranking system, which, by design, may result in more substances being classified as high priority, mainly when scoring criteria emphasize hazardous properties and exposure characteristics. This could dilute the focus of the HBM program by requiring resources to be allocated to a broader range of chemicals, some of which may have less immediate relevance. To overcome this limitation in the later stages of this project, a pilot study with a limited number of recruited participants (30) will be organized to test several aspects of the newly established program, including one related to the prevalence of chemicals. The Human Biomonitoring Council of Latvia has approved including all 30 chemical substances assessed as high priority, or their metabolites, in the pilot study, depending on the availability of laboratories and the capacity to analyze these substances. The pilot study will also incorporate quality assurance measures, such as participation in proficiency testing programs like the German External Quality Assessment Scheme (GEQUAS), to ensure the reliability and comparability of the results.

In addition to the possible high number of chemical substances being classified as high priority, one of the potential limitations of the six-step approach used in Latvia is the risk of excluding some emerging chemicals during the shortlisting process in step 3. While efforts were made to incorporate a wide range of substances through literature reviews and consultations with national authorities, chemicals with limited data or recent identification may not have been adequately considered. This highlights the broader challenge of addressing the dynamic nature of chemical production and use. Future iterations could benefit from more robust mechanisms, such as periodic reviews of the HBM program and especially emerging contaminants, enhanced data collection, and closer collaboration with international initiatives to ensure inclusivity, consistency, and adaptability in the prioritization process.

To address these issues, future refinements to the methodology could include more robust mechanisms for periodic review and inclusion of emerging contaminants, such as leveraging real-time data from international chemical monitoring initiatives. Additionally, adjusting the scoring thresholds or incorporating a weighting system to further distinguish between chemicals based on national priorities and resource constraints could help optimize the ranking process. These enhancements would ensure that the methodology remains dynamic, inclusive, and better aligned with both scientific and practical considerations. Despite the above-mentioned challenges, the methodology developed in this study offers a scalable model for other countries seeking to establish their own HBM programs. Future efforts should focus on strengthening data collection mechanisms, improving regulatory frameworks, and fostering international collaboration to ensure a more comprehensive and harmonized approach to human biomonitoring.

## 5. Conclusions

This study prioritized chemical substances for Latvia’s HBM program using the adapted Hanlon methodology, identifying 30 high-priority substances. The approach considered public health significance, national priorities, and public interest, while addressing data gaps and aligning with European frameworks. Hazardous properties were a critical component in the prioritization process, ensuring that substances with significant risks to public health and the environment were adequately identified. The resulting list of prioritized substances provides a solid foundation for implementing the HBM4LV program, which will monitor chemical exposure and support evidence-based policy making. Despite limitations, such as resource constraints and incomplete data, this methodology offers a scalable model for future prioritization efforts. Continued development and collaboration will strengthen the program’s effectiveness in protecting public health and the environment.

## Figures and Tables

**Table 1:** Adapted Hanlon Method.

Component	Area	Possible Points	Use of Points
A	Problem size (percentage of exposed population)	1	<1.0%
		2–3	1–9.9% OR no data
		4–5	10–24.9%
		6	>25%
B	Hazardous properties: (carcinogenicity, mutagenicity, reproductive toxicity, developmental toxicity, endocrine activity, systemic toxicity after repeated exposure (STOT RE), neurotoxicity, immunotoxicity, respiratory sensitization, skin sensitization)	1	Low grade
		3	Average grade
		6	High grade
C	Exposure characteristics (persistency and/or bioaccumulation potential, sales in the EU or, where possible, sales in Latvia (tonnes per year), exposure routes, passage of placental barrier, exposed population, level of concern of the exposure)	1	Low level
		3	Average level
		6	High level
D	National significance	0	No
		1	Yes
E	Public interest	0	No
		1	Yes

**Table 2:** A **c**ombined list of substances ranked from highest score to lowest for the chemicals classified with high priority for inclusion in the HBM4LV program suggested by experts.

	Group (Subgroup) of Substances	Name of Substance	CAS Number	Points A	Points B	**Points C**	**Points D**	**Points E**	**Total Score**
1	Pesticides	Glyphosate	1071-83-6	60	20.4	35.1	15	15	145.5
2	Persistent organic pollutants (per- and poly-fluoroalkyl substances)	Perfluorooctane sulfonate	1763-23-1	60	18.9	24.9	15	15	133.8
3	Others (phthalates)	Bis(2-ethylhexyl) phthalate/Di(2-ethylhexyl) phthalate (DEHP)	117-81-7	60	9.9	30.0	15	15	129.9
4	Persistent organic pollutants (polychlorinated biphenyls)	Polychlorinated biphenyls (PCBs)	1336-36-3	60	15.0	23.4	15	15	128.4
5	Persistent organic pollutants (polycyclic aromatic hydrocarbons)	Benzo(a)pyrene	50-32-8	60	12.0	25.8	15	15	127.8
6	Others (parabens)	Methylparaben	99-76-3	60	10.5	24.5	15	15	125.0
7	Others (bisphenols)	Bisphenol A	80-05-7	60	15.0	20.1	15	15	125.1
8	Others (phthalates)	Diisobutyl phthalate (DiBP)	84-69-5	60	10.0	24.9	15	15	124.9
9	Others (parabens)	Ethylparaben	120-47-8	60	9.0	24.9	15	15	123.9
10	Persistent organic pollutants (flame retardants)	Decabromodiphenyl ether; Bis(pentabromophenyl) ether	1163-19-5	60	6.0	27.6	15	15	123.6
11	Persistent organic pollutants (flame retardants)	Octabromodiphenyl ether	32536-52-0	60	7.5	25.8	15	15	123.3
12	Others (phthalates)	Di-n-butyl phthalate (DnBP)	84-74-2	60	10.5	22.5	15	15	123.0
13	Others (phthalates)	Diethyl phthalate (DEP)	84-66-2	60	8.1	22.5	15	15	120.6
14	Others (phthalates)	Butyl benzyl phthalate (BBzP)	85-68-7	60	9.9	20.7	15	15	120.6
15	Others (phthalates)	Diisohexyl phthalate	71850-09-4/ 607-737-00-2	60	18.0	26.7	0	15	119.7
16	Others (phthalates)	Diisononyl cyclohexane-1,2-dicarboxylate (HEXAMOLL)	166412-78-8	60	9.0	20.7	15	15	119.7
17	Persistent organic pollutants (flame retardants)	2,2′,6,6′-tetrabromo-4,4′-isopropylidenediphenol	79-94-7	60	13.5	30.0	0	15	118.5
18	Others (phthalates)	Diisononyl phthalate (DiNP)	28553-12-0/ 41375-91-1/ 68515-48-0/ 105009-97-0	60	3.0	24.9	15	15	117.9
19	Others (parabens)	Butylparaben	94-26-8	60	14.1	27.6	15	0	116.7
20	Others (phthalates)	Dicyclohexyl phthalate (DCHP)	84-61-7	60	19.5	21.6	0	15	116.1
21	Persistent organic pollutants (flame retardants)	Hexabromocyclododecane	25637-99-4	60	1.5	24.5	15	15	116.0
22	Pesticides	Acetamiprid	135410-20-7	60	4.5	35.1	0	15	114.6
23	Persistent organic pollutants (polycyclic aromatic hydrocarbons)	Benzo(k)fluoranthene	207-08-9	60	13.5	25.8	15	0	114.3
24	Persistent organic pollutants (polycyclic aromatic hydrocarbons)	Dibenzo(ah)anthracene	53-70-3	60	13.5	25.8	15	0	114.3
25	Persistent organic pollutants (polycyclic aromatic hydrocarbons)	Benzo(b)fluoranthene	205-99-2	60	13.5	25.8	15	0	114.3
26	Persistent organic pollutants (polycyclic aromatic hydrocarbons)	Benzo(e)pyrene	192-97-2	60	12.6	25.8	15	0	113.4
27	Others (phthalates)	Diisodecyl phthalate (DiDP)	26761-40-0/ 105009-98-1/ 1341-39-5	60	0.9	22.5	15	15	113.4
28	Others (parabens)	Propylparaben	94-13-3	60	10.5	27.6	15	0	113.1
29	Persistent organic pollutants (polycyclic aromatic hydrocarbons)	Indeno(1,2,3-cd)pyrene	193-39-5	60	11.1	25.8	15	0	111.9
30	Others (phthalates)	2-[(2-methyl-1-oxoallyl)oxy]ethyl hydrogen 3-chloro-2-hydroxypropylphthalate	54380-33-5	60	11.1	24.9	0	15	111.0
31	Others (phthalates)	2-acryloyloxyethyl hydrogen phthalate	30697-40-6	60	11.1	24.9	0	15	111.0
32	Persistent organic pollutants (polycyclic aromatic hydrocarbons)	Benzo(ghi)perylene	191-24-2	60	9.6	25.8	15	0	110.4
33	Persistent organic pollutants (per- and poly-fluoroalkyl substances)	Perfluorooctanoic acid	335-67-1	30	21.9	28.2	15	15	110.1
34	Others (phthalates)	1,2-Benzenedicarboxylic acid, di-C6-8-branched alkyl esters, C7-rich	71888-89-6	60	10.0	24.9	0	15	109.9
35	Persistent organic pollutants (polycyclic aromatic hydrocarbons)	Anthracene	120-12-7	60	9.9	24.9	15	0	109.8
36	Others (phthalates)	Di(2-propylheptyl) phthalate (DPHP)	53306-54-0	60	9.9	9.9	15	15	109.8
37	Others (phthalates)	Bis(2-ethylhexyl) terephthalate (DEHTP)	6422-86-2/1264916-12-2/144981-82-8)	60	9.9	9.9	15	15	109.8
38	Others (phthalates)	Di-n-octyl phthalate (DnOP)	117-84-0	60	12.9	20.7	0	15	108.6
39	Persistent organic pollutants (polycyclic aromatic hydrocarbons)	Pyrene	129-00-0	60	6.6	24.9	15	0	106.5
40	Others (phthalates)	Bis(2-ethylhexyl) tetrabromophthalate	118817-35-9	60	10.5	21.0	0	15	106.5
41	Others (acrylamide)	Acrylamide	79-06-1	30	24.0	21.7	15	15	105.7
42	Others (phthalates)	Di-n-pentyl phthalate (DnPeP)	131-18-0	60	10.0	20.7	0	15	105.7
43	Others (phthalates)	Di-isopentyl phthalate (DiPeP)	605-50-5	60	10.0	20.7	0	15	105.7
44	Others (phthalates)	Di-C7-11-(linear and branched)-alkyl phthalate (DHNUP)	68515-42-4	60	10.0	20.7	0	15	105.7
45	Others (phthalates)	Di-n-hexyl phthalate (DnHP)	84-75-3	60	10.0	20.7	0	15	105.7
46	Others (phthalates)	Di(methoxyethyl) phthalate (DMEP)	117-82-8	60	10.0	20.7	0	15	105.7
47	Others (phthalates)	Mono-1-tert-butyl-3-methylbutyl phthalate	109591-02-8	60	6.0	21.6	0	15	102.6
48	Persistent organic pollutants (flame retardants)	Pentabromodiphenyl ether	32534-81-9	60	12.0	0	15	15	102.0
49	Others (phthalates)	(2-ethylhexyl) hydrogen phthalate	4376-20-9	60	5.0	20.7	0	15	100.7
50	Others (phthalates)	Mono-(1,2,2-trimethylpropyl) phthalate	84489-36-1	60	3.9	21.6	0	15	100.5
51	Metals	Mercury	7439-97-6	30	17.4	21.6	15	15	99.0
52	Metals	Lead	7439-92-1	40	21.0	20.7	15	0	96.7

**Table 3:** A **c**ombined list of substances ranked from highest score to lowest for the chemicals classified with high priority for inclusion in the HBM4LV program approved by the Human Biomonitoring Council of Latvia.

	Group (Subgroup) of Substances	Name of Substance	CAS Number	Points A	Points B	Points C	Points D	Points E	**Total Score**
1	Pesticides	Glyphosate	1071-83-6	60	20.4	35.1	15	15	145.5
2	Persistent organic pollutants (per- and poly-fluoroalkyl substances)	Perfluorooctane sulfonate	1763-23-1	60	18.9	24.9	15	15	133.8
3	Others (phthalates)	Bis(2-ethylhexyl) phthalate/Di(2-ethylhexyl) phthalate (DEHP)	117-81-7	60	9.9	30.0	15	15	129.9
4	Persistent organic pollutants (polycyclic aromatic hydrocarbons)	Benzo(a)pyrene	50-32-8	60	12.0	25.8	15	15	127.8
5	Others (bisphenols)	Bisphenol A	80-05-7	60	15.0	20.1	15	15	125.1
6	Others (phthalates)	Diisobutyl phthalate (DiBP)	84-69-5	60	10.0	24.9	15	15	124.9
7	Persistent organic pollutants (flame retardants)	Decabromodiphenyl ether; Bis(pentabromophenyl) ether	1163-19-5	60	6.0	27.6	15	15	123.6
8	Persistent organic pollutants (flame retardants)	Octabromodiphenyl ether	32536-52-0	60	7.5	25.8	15	15	123.3
9	Others (phthalates)	Di-n-butyl phthalate (DnBP)	84-74-2	60	10.5	22.5	15	15	123.0
10	Others (phthalates)	Diisohexyl phthalate	71850-09-4/ 607-737-00-2	60	18.0	26.7	0	15	119.7
11	Persistent organic pollutants (flame retardants)	2,2′,6,6′-tetrabromo-4,4′-isopropylidenediphenol	79-94-7	60	13.5	30.0	0	15	118.5
12	Others (phthalates)	Dicyclohexyl phthalate (DCHP)	84-61-7	60	19.5	21.6	0	15	116.1
13	Persistent organic pollutants (flame retardants)	Hexabromocyclododecane	25637-99-4	60	1.5	24.5	15	15	116.0
14	Pesticides	Acetamiprid	135410-20-7	60	4.5	35.1	0	15	114.6
15	Persistent organic pollutants (polycyclic aromatic hydrocarbons)	Benzo(k)fluoranthene	207-08-9	60	13.5	25.8	15	0	114.3
16	Persistent organic pollutants (polycyclic aromatic hydrocarbons)	Dibenzo(ah)anthracene	53-70-3	60	13.5	25.8	15	0	114.3
17	Persistent organic pollutants (polycyclic aromatic hydrocarbons)	Benzo(b)fluoranthene	205-99-2	60	13.5	25.8	15	0	114.3
18	Persistent organic pollutants (polycyclic aromatic hydrocarbons)	Benzo(e)pyrene	192-97-2	60	12.6	25.8	15	0	113.4
19	Persistent organic pollutants (per- and poly-fluoroalkyl substances)	Perfluorooctanoic acid	335-67-1	30	21.9	28.2	15	15	110.1
20	Others (phthalates)	1,2-Benzenedicarboxylic acid, di-C6-8-branched alkyl esters, C7-rich	71888-89-6	60	10.0	24.9	0	15	109.9
21	Others (phthalates)	Bis(2-ethylhexyl) tetrabromophthalate	118817-35-9	60	10.5	21.0	0	15	106.5
22	Others (acrylamide)	Acrylamide	79-06-1	30	24.0	21.7	15	15	105.7
23	Others (phthalates)	Di-n-pentyl phthalate (DnPeP)	131-18-0	60	10.0	20.7	0	15	105.7
24	Others (phthalates)	Di-isopentyl phthalate (DiPeP)	605-50-5	60	10.0	20.7	0	15	105.7
25	Others (phthalates)	Di-C7-11-(linear and branched)-alkyl phthalate (DHNUP)	68515-42-4	60	10.0	20.7	0	15	105.7
26	Others (phthalates)	Di-n-hexyl phthalate (DnHP)	84-75-3	60	10.0	20.7	0	15	105.7
27	Others (phthalates)	Di(methoxyethyl) phthalate (DMEP)	117-82-8	60	10.0	20.7	0	15	105.7
28	Persistent organic pollutants (flame retardants)	Pentabromodiphenyl ether	32534-81-9	60	12.0	0	15	15	102.0
29	Metals	Mercury	7439-97-6	30	17.4	21.6	15	15	99.0
30	Metals	Lead	7439-92-1	40	21.0	20.7	15	0	96.7

## Data Availability

No new data were created or analyzed in this study. Data sharing does not apply to this article.

## Appendix A

**Table A1:** Example of using the adapted Hanlon method (Acetamiprid, CAS number 135410-20-7).

Component	Number of Points	Weights	Results	Comments, References
A	6	10	60	32.84% (n = 132) of samples tested during HBM4EU Survey on PEstiCIde Mixtures in Europe ^1^
B	0.15	30	4.5	Carcinogenicity—0 points Mutagenicity—0 points Reproductive toxicity—3 points ^2^ Developmental toxicity—3 points ^2^ Endocrine activity—3 points ^3^ STOT RE—0 points Neurotoxicity—0 points Immunotoxicity—0 points Respiratory sensitizer—0 points Skin sensitizer—0 points Total 9 points 9/60 = 0.15
C	1.17	30	35.1	Persistency and bioaccumulation potential—6 points ^4^ Sales in Latvia (tonnes)—1 point ^5^ Exposure routes—6 points ^6^ Passage of placental barrier—6 points ^7^ Exposed population—15 points ^8^ Level of concern of the exposure—8 points ^9^ Total 42 points 42/36 = 1.17
D	0	15	0	
E	1	15	15	
Total			114.6	

^1.^ Data from Vlaanderen J. et al. [24]. ^2.^ Summary of Classification and Labeling (Acetamiprid). ECHA. Available online: https://echa.europa.eu/lv/information-on-chemicals/cl-inventory-database/-/discli/details/26276 (assessed on 12 April 2024). ^3.^ TEDX. The Endocrine Disruption Exchange. Available online: https://endocrinedisruption.org/interactive-tools/tedx-list-of-potential-endocrine-disruptors/search-the-tedx-list (assessed on 18 April 2024). ^4.^ Ma X. et al. Long-Term Exposure to Neonicotinoid Insecticide Acetamiprid at Environmentally Relevant Concentrations Impairs Endocrine Functions in Zebrafish: Bioaccumulation, Feminization, and Transgenerational Effects. Environmental Science&Technology 2022, 6;56(17):12494-12505. doi: 10.1021/acs.est.2c04014. Zuščíková, L. et al. Screening of Toxic Effects of Neonicotinoid Insecticides with a Focus on Acetamiprid: A Review. Toxics 2023, 11, 598. https://doi.org/10.3390/toxics11070598. ^5.^ The Statistics of Plant Protection Product Sales [25]. ^6.^ United States Environmental Protection Agency. Pesticide Fact Sheet. Acetamiprid. Available online: https://www3.epa.gov/pesticides/chem_search/reg_actions/registration/fs_PC-099050_15-Mar-02.pdf (assessed on 18 April 2024). ^7.^ Zhang X. Neonicotinoid Insecticides and Their Metabolites Can Pass through the Human Placenta Unimpeded. Ecotoxicology and Public Health 2022, 56 (23). doi: 10.1021/acs.est.2c06091. ^8.^ Employees, general population: CLH Report for Acetamiprid. ECHA. Available online: https://echa.europa.eu/documents/10162/2c6b3db4-7884-b0c9-3109-677ebd3d5b01 (assessed on 15 April 2024); United States Environmental Protection Agency. Pesticide Face Sheet. Acetamiprid. Available online: https://www3.epa.gov/pesticides/chem_search/reg_actions/registration/fs_PC-099050_15-Mar-02.pdf (assessed on 17 April 2024). Vulnerable groups—children up to 6, pregnant women: United States Environmental Protection Agency. Pesticide Face Sheet. Acetamiprid. Available online: https://www3.epa.gov/pesticides/chem_search/reg_actions/registration/fs_PC-099050_15-Mar-02.pdf (assessed on 18 April 2024). ^9.^ Acetamiprid is the most commonly found insecticide in Latvian honey and pollen samples; moreover, in some honey samples, its concentration has exceeded the maximum residue level (0.05 mg/kg). Ozols N. et al. Pesticide Contamination of Honey-Bee-Collected Pollen in the Context of the Landscape Composition in Latvia. Toxics 2024, 12(12):862. doi: 10.3390/toxics12120862.

**Table A2:** List of all chemicals assessed with the adapted Hanlon methodology.

Group of Substances	Substance	CAS Number	Component Scores					**Total Score**	**Priority**
			A	B	C	D	**E**		
Pesticides									
	Glyphosate	1071-83-6	60	20.4	35.1	15	15	145.5	High
	Acetamiprid	135410-20-7	60	4.5	35.1	0	15	114.6	High
	Bentazone	25057-89-0	30	6.6	24.3	15	15	90.9	Average
	Prosulfocarb	52888-80-9	30	9	21.6	15	15	90.6	Average
	MCPA	3653-48-3	30	2.4	20.7	15	15	83.1	Average
	Chlorpropham	101-21-3	60	4.5	18.3	0	0	82.8	Average
	Organophosphate pesticides								
	Dimethoate	60-51-5	30	12.9	20.1	15	0	78.0	Average
	Pirimiphos-methyl	29232-93-7	40	13.5	21.6	0	0	75.1	Average
	Metazachlor	67129-08-2	30	9.9	18.3	15	0	73.2	Low
	Chlormequat chloride	999-81-5	30	4.5	22.5	15	0	72.0	Low
	Tebuconazole	107534-96-3	30	3.9	20.1	15	0	69.0	Low
	Boscalid	188425-85-6	50	2.1	16.8	0	0	68.9	Low
	Pendimethalin	40487-42-1	30	3.9	17.4	15	0	66.3	Low
	Dimetachlor	50563-36-5	30	3.9	10.8	15	0	59.7	Low
	Diflufenican	83164-33-4	30	0.6	12.6	15	0	58.2	Low
	Quinmerak	90717-03-6	30	0.9	9.9	15	0	55.8	Low
Persistent organic pollutants									
	Flame retardants								
	Decabromodiphenyl ether; Bis(pentabromophenyl) ether	1163-19-5	60	6	27.6	15	15	123.6	High
	Octabromodiphenyl ether	32536-52-0	60	7.5	25.8	15	15	123.3	High
	2.2′.6.6′-tetrabromo-4.4′-isopropylidenediphenol	79-94-7	60	13.5	30	0	15	118.5	High
	Hexabromocyclododecane	25637-99-4	60	1.5	24.5	15	15	116	High
	Pentabromodiphenyl ether	32534-81-9	60	12	0	15	15	102.0	High
	Tris(2-chloroethyl)phosphate	115-96-8	20	12	0	15	0	47.0	
	ris[2-chloro-1-(chloromethyl)ethyl]phosphate	13674-87-8	20	9	0	15	0	44.0	
	4.4′-dibromobiphenyl	92-86-4	20	3	0	0	0	23.0	
	2.2′-dibromobiphenyl	2052-07-5	20	1.5	0.9	0	0	22.4	
	3-bromobiphenyl	2113-57-7	20	1.5	0.9	0	0	22.4	
	3.3′-dibromobiphenyl	16400-51-4	20	1.5	0	0	0	21.5	
	Polycyclic aromatic hydrocarbons (PAHs)								
	Benzo(a)pyrene	50-32-8	60	12	25.8	15	15	127.8	High
	Benzo(k)fluoranthene	207-08-9	60	13.5	25.8	15	0	114.3	High
	Dibenzo(ah)anthracene	53-70-3	60	13.5	25.8	15	0	114.3	High
	Benzo(e)pyrene	192-97-2	60	12.6	25.8	15	0	113.4	High
	Benzo(b)fluoranthene	205-99-2	60	13.5	25.8	15	0	114.3	High
	Indeno(1.2.3-cd)pyrene	193-39-5	60	11.1	25.8	15	0	111.9	High
	Benzo(ghi)perylene	191-24-2	60	9.6	25.8	15	0	110.4	High
	Anthracene	120-12-7	60	9.9	24.9	15	0	109.8	High
	Pyrene	129-00-0	60	6.6	24.9	15	0	106.5	High
	Benzo(a)anthracene	129-00-0	20	13.5	24.9	15	0	73.4	Low
	Chrysene	218-01-9	20	9.3	25.8	15	0	70.1	Low
	Fluoranthene	206-44-0	20	6.6	25.8	15	0	67.4	Low
	Naphthalene	91-20-3	20	7.5	30	0	0	57.5	Low
	Acenaphthene	83-32-9	20	6.9	25.8	0	0	52.7	
	Phenanthrene	85-01-8	20	6.6	25.8	0	0	52.4	
	Acenaphthylene	208-96-8	20	6	25.8	0	0	51.8	
	Fluorene	86-73-7	20	6.6	24.9	0	0	51.5	
	Per- and polyfluoroalkyl substances (PFASs)								
	Perfluorooctane sulfonate	1763-23-1	60	18.9	24.9	15	15	133.8	High
	Perfluorooctanoic acid	335-67-1	30	21.9	28.2	15	15	110.1	High
	Perfluorononanoic acid	375-95-1	10	15.9	28.2	15	15	84.1	Average
	Perfluorohexane sulfonic acid	355-46-4	10	14.4	24.9	15	15	79.3	Average
	Polychlorinated biphenyls. dioxin-like biphenyls and biphenyls								
	Polychlorinated biphenyls (PCB)	1336-36-3	60	15	23.4	15	15	128.4	High
	Biphenyl-3.3′.4.4′-tetrayltetraammonium tetrachloride	7411-49-6	20	15.9	2.4	0	0	38.3	
	Biphenyl-4-aminium chloride	2113-61-3	20	15.9	2.4	0	0	38.3	
	4-nitrobiphenyl	92-93-3	20	14.1	2.4	0	0	36.5	
	Biphenyl-3.3′.4.4′-tetrayltetraamine	91-95-2	20	12	2.4	0	0	34.4	
	Biphenyl-4-ylamine	92-67-1	20	11.1	2.4	0	0	33.5	
	Dioxins								
	1.2.3.6.7.8-Hexachlorodibenzo-p-dioxin	57653-85-7	60	9.9	21.6	0	0	91.5	Average
	1.2.3.4.6.7.8-Heptachlorodibenzo-p-dioxin	35822-46-9	60	9.9	9.6	0	0	79.5	Average
	2.3.7.8-Tetrachlorodibenzo[b.e][1.4]dioxin	1746-01-6	30	12.9	26.7	0	0	69.6	Low
	1.2.3.4.7.8-Hexachlorodibenzodioxin	39227-28-6	30	12	26.7	0	0	68.7	Low
	1.2.3.7.8.9-Hexachlorodibenzo-p-dioxin	19408-74-3	30	9.9	26.7	0	0	66.6	Low
	1.2.3.4.6.7.8.9-Octachlorodibenzo-p-dioxin	3268-87-9	30	9.9	26.7	0	0	66.6	Low
	1.2.3.7.8-Pentachlorodibenzo-p-dioxin	40321-76-4	30	9	26.7	0	0	65.7	Low
	Furans								
	2.3.4.7.8-Pentachlorodibenzofuran	57117-31-4	60	6.9	11.7	0	0	78.6	Average
	Furan	110-00-9	30	7.5	16.8	0	0	54.3	
	1.2.3.7.8-Pentachlorodibenzofuran	57117-41-6	30	7.5	16.8	0	0	54.3	
	1.2.3.4.7.8-Hexachlorodibenzofuran	70648-26-9	30	4.5	16.8	0	0	51.3	
	Dibenzofuran. 2.3.7.8-tetrachloro-	51207-31-9	30	5.4	15.9	0	0	51.3	
	6-isopropyl-1.3.8-trichlorodibenzofuran	125652-13-3	30	3.6	16.8	0	0	50.4	
	6-Methyl-1.3.8-trichlorodibenzofuran	118174-38-2	30	3.6	16.8	0	0	50.4	
	6-Methyl-2.3.4.8-tetrachlorodibenzofuran	139883-51-5	30	3.6	15	0	0	48.6	
	1.2.3.4.6.7.8.9-Octachlorodibenzofuran	39001-02-0	30	2.4	15	0	0	47.4	
Metals									
	Mercury	7439-97-6	30	17.4	21.6	15	15	99.0	High
	Lead	7439-92-1	40	21	20.7	15	0	96.7	High
	Cadmium	7440-43-9	30	21	24	15	0	90.0	Average
	Arsenic	7440-38-2	20	17.1	20.1	15	0	72.2	Low
	Nickel	7440-02-0	20	16.5	18.3	15	0	69.8	Low
	Chromium	7440-47-3/16065-83-1/18540-29-9	20	13.5	7.5	15	0	56.0	Low
	Zinc	7440-66-6	20	2.1	15.9	15	0	53.0	
	Vanadium	7440-62-2	20	17.1	12.6	0	0	49.7	
	Cobalt	7440-48-4	10	18	11.7	0	0	39.7	
Others									
	Acrylamide	79-06-1	30	24	21.7	15	15	105.7	High
	Bisphenols								
	Bisphenol A	80-05-7	60	15	20.1	15	15	125.1	High
	Bisphenol S	80-09-1	60	3	2.4	15	0	80.4	Average
	Bisphenol F	2467-02-9	60	6	11.7	0	0	77.7	Average
	Bisphenol M	13595-25-0	20	9	11.7	0	0	40.7	
	Bisphenol AF	1478-61-1	20	9	7.5	0	0	36.5	
	DHDPE. p.p′-oxybisphenol	1965-09-9	20	4.5	6.5	0	0	31.0	
	Tetrabromo-bisphenol S	39635-79-5	20	3	6.6	0	0	29.6	
	Bisphenol Z bis(chloroformate)	91174-67-3	20	3	6.6	0	0	29.6	
	Bisphenol fluorenone	3236-71-3	20	1.5	6.6	0	0	28.1	
	Phatalates								
	Bis(2-ethylhexyl) phthalate/Di(2-ethylhexyl) phthalate (DEHP)	117-81-7	60	9.9	30	15	15	129.9	High
	Diisobutyl phthalate (DiBP)	84-69-5	60	10	24.9	15	15	124.9	High
	Di-n-butyl phthalate (DnBP)	84-74-2	60	10.5	22.5	15	15	123.0	High
	Diethyl phthalate (DEP)	84-66-2	60	8.1	22.5	15	15	120.6	High
	Butyl benzyl phthalate (BBzP)	85-68-7	60	9.9	20.7	15	15	120.6	High
	Diisohexyl phthalate	71850-09-4/607-737-00-2	60	18	26.7	0	15	119.7	High
	Diisononyl cyclohexane-1.2-dicarboxylate (HEXAMOLL)	166412-78-8	60	9	20.7	15	15	119.7	High
	Diisononyl phthalate (DiNP)	28553-12-0/41375-91-1/68515-48-0/105009-97-0	60	3	24.9	15	15	117.9	High
	Dicyclohexyl phthalate (DCHP)	84-61-7	60	19.5	21.6	0	15	116.1	High
	Diisodecyl phthalate (DiDP)	26761-40-0/105009-98-1/1341-39-5	60	0.9	22.5	15	15	113.4	High
	2-[(2-methyl-1-oxoallyl)oxy]ethyl hydrogen 3-chloro-2-hydroxypropylphthalate	54380-33-5	60	11.1	24.9	0	15	111.0	High
	2-acryloyloxyethyl hydrogen phthalate	30697-40-6	60	11.1	24.9	0	15	111.0	High
	1.2-Benzenedicarboxylic acid. di-C6-8-branched alkyl esters. C7-rich	71888-89-6	60	10	24.9	0	15	109.9	High
	Di(2-propylheptyl) phthalate (DPHP)	53306-54-0	60	9.9	9.9	15	15	109.8	High
	Bis(2-ethylhexyl) terephthalate (DEHTP)	6422-86-2/1264916-12-2/144981-82-8)	60	9.9	9.9	15	15	109.8	High
	Di-n-octyl phthalate (DnOP)	117-84-0	60	12.9	20.7	0	15	108.6	High
	Bis(2-ethylhexyl) tetrabromophthalate	118817-35-9	60	10.5	21	0	15	106.5	High
	Di-n-pentyl phthalate (DnPeP)	131-18-0	60	10	20.7	0	15	105.7	High
	Di-isopentyl phthalate (DiPeP)	605-50-5	60	10	20.7	0	15	105.7	High
	Di-C7-11-(linear and branched)-alkyl phthalate (DHNUP)	68515-42-4	60	10	20.7	0	15	105.7	High
	Di-n-hexyl phthalate (DnHP)	84-75-3	60	10	20.7	0	15	105.7	High
	Di(methoxyethyl) phthalate (DMEP)	117-82-8	60	10	20.7	0	15	105.7	High
	Mono-1-tert-butyl-3-methylbutyl phthalate	109591-02-8	60	6	21.6	0	15	102.6	High
	(2-ethylhexyl) hydrogen phthalate	4376-20-9	60	5	20.7	0	15	100.7	High
	Mono-(1.2.2-trimethylpropyl) phthalate	84489-36-1	60	3.9	21.6	0	15	100.5	High
	Triclosan	3380-34-5	60	4.5	15	0	0	79.5	Average
	Parabens								
	Methylparaben	99-76-3	60	10.5	24.5	15	15	125.0	High
	Ethylparaben	120-47-8	60	9	24.9	15	15	123.9	High
	Butylparaben	94-26-8	60	14.1	27.6	15	0	116.7	High
	Propylparaben	94-13-3	60	10.5	27.6	15	0	113.1	High
	Benzylparaben	94-18-8	60	3.9	13.2	15	0	92.1	Average
	Isobutylparaben	4247-02-3	40	0	12.6	15	0	67.6	Low
	Heptylparaben	1085-12-7	40	3.6	13.2	0	0	56.8	Low
	Isopentylparaben	6521-30-8	40	3.6	13.2	0	0	56.8	Low
	Isopropylparaben	4191-73-5	40	3.6	13.2	0	0	56.8	Low
	Nonylparaben	38713-56-3	40	3.6	13.2	0	0	56.8	Low
	Phenylparaben	17696-62-7	40	3.6	7.5	0	0	51.1	
	Sodium paraben	114-63-6	20	0.6	13.2	0	0	33.8	
	Hexamidine paraben	93841-83-9	20	0	13.2	0	0	33.2	
	Potassium paraben	16782-08-4	20	0	13.2	0	0	33.2	
